# Starch, gallic acid, their inclusion complex and their effects in diabetes and other diseases—A review

**DOI:** 10.1002/fsn3.3208

**Published:** 2022-12-28

**Authors:** Sayoni Nag, Suman Majumder

**Affiliations:** ^1^ Department of Biotechnology Brainware University Barasat India

**Keywords:** Diabetes, Diseases, Gallic acid, Inclusion complex, Starch

## Abstract

Starch is the most important energy‐providing component of food. It is useful for maintaining the structural and rheological consistency of food, ad thus, in turn, is responsible for maintaining the freshness of food. Polyphenols are present in plant products in huge amounts as secondary metabolites. Gallic acid, one of the potent plant polyphenols, has been reported to have excellent anti‐inflammatory, antioxidative, anticarcinogenic, microbicidal, and antidiabetic properties. Till date, very few articles on the starch–polyphenol inclusion complex are present. Quite a few hypotheses have been proposed as to how the formation of an inclusion complex of starch with polyphenol can slower the digestion or the hydrolysis of starch. The efficient qualities of starch–polyphenol systems, such as reduced starch digestion, lower blood glucose and preserving food freshness, have formed a necessity for investigation in this area. The focus of this review centers on the recent research on starch–polyphenol interactions and starch–gallic acid inclusion complexes in native and extruded food systems, as well as how the production of these complexes can aid in the treatment of diseases, particularly diabetes mellitus.

## Graphical abstract

The three types of approaches (Approach A, Approach B and Approach C) to improve insulin sensitivity and protect ß cells of the pancreas.

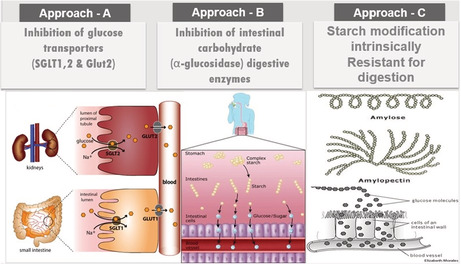

## 1 INTRODUCTION

Postprandial glucose (PPG) is a key factor in the etiology of T2D and other metabolic disorders. Patients with diabetes mellitus may experience postprandial glycemic excursions as a result of dietary variables, particularly carbohydrates (Alssema et al., 2015). Many common dietary items, such as grains, roots, and tubers contain a significant amount of starch, a high‐glycemic carbohydrate substance. The type of food consumed and the starch composition affect the meal's glycemic index (Omage & Omage, 2017). Chronic consumption of high GI products can lead to an increase in PPG.

**FIGURE 1:**
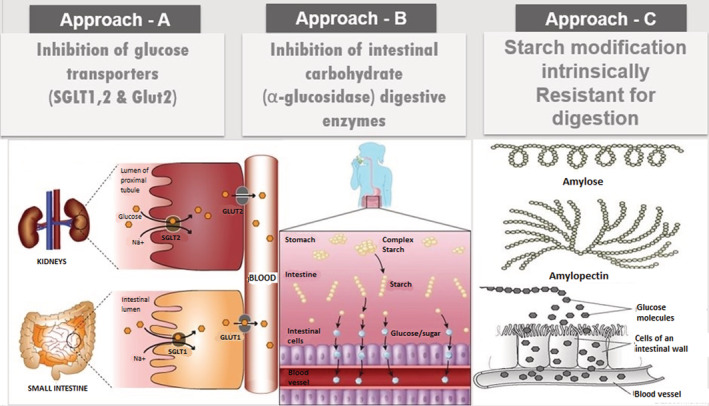
The three types of approaches (Approach A, Approach B and Approach C) to improve insulin sensitivity and protect β‐cells of the pancreas.

The standard approaches to control PPG are to (A) inhibit glucose transporters (SGLT1,2 & Glut2) (Forester et al., 2012); (B) inhibition of salivary and intestinal carbohydrate (α‐amylase and α‐glucosidase) digestive enzymes (Malunga et al., 2016); (C) consume modified starch intrinsically resistant toward digestion (Mah et al., 2018). Apart from these, there are interventions to improve insulin sensitivity and protect β‐cells of the pancreas (Rorsman & Ashcroft, 2018) (Figure 1).

For a while, synthetic medications such as sulfonyl ureas and biguanides may be successful in regulating blood sugar levels, but they come with a risk of adverse effects including hypoglycemia, nausea, vomiting, cholestatic jaundice, and other medical issues (Feingold, 2000). The majority of type 2 DM patients initially respond well to medications that lower blood glucose levels (BGLs), but after some time, 20% of them develop resistance and do not benefit from them (Berbudi et al., 2020). Due to the decline in regular diet and exercise, the progression of beta cell failure, drug resistance, and other health issues, patients may also not fully respond to these drugs (Tomic et al., 2022). So, there is continuous effort in search of interventions which are either staple foods or sourced from natural plant materials to control diabetes mellitus type II in a more effective but safe way.

Most patients ultimately need insulin treatment (Berbudi et al., 2020). The traditional approach to control diabetes mellitus comprises lifestyle dietary involvements, work out, and a diversity of hypoglycemic herbs and herbal modifications. Many of these herbs are rich in polyphenols, such as tannins, gallic acid, and gallates (Wang, Alkhalidy, & Liu, 2021).

**FIGURE 2:**
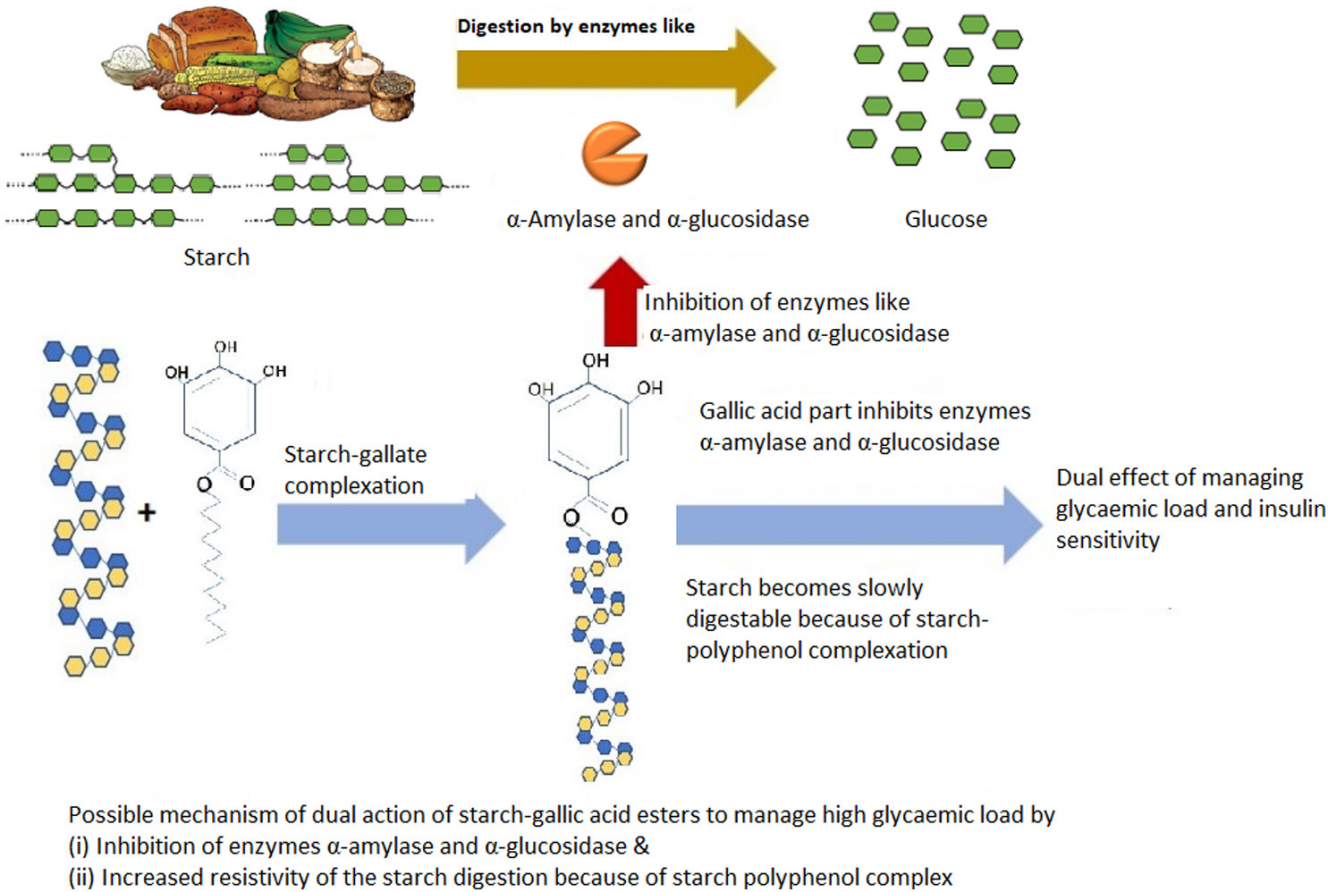
The possible structures and structural modifications of formation of starch gallic acid inclusion complexes.

Rice, grains, roots, and tubers, which are traditional staple food sources, as well as the numerous products made from them are sources of starch, one of the most significant glycemic carbohydrate components in foods (Bao, 2017). The glycemic index of starchy foods is based on how quickly glucose is digested and absorbed in the small intestine. From a nutritional standpoint, starch has been divided into three categories: quickly digested starch (RDS) (Chung et al., 2011), slowly digestible starch (SDS) (Miao et al., 2015), and resistant starch (RS) to further explain its digestive qualities in food products (Zhang & Hamaker, 2009). RDS is quickly digested and fast absorbed in the duodenum and proximal regions of the small intestine, causing a rapid rise in blood glucose that is typically followed by a condition known as hypoglycemia (Aller et al., 2011). RDS is found in foods like gelatinized waxy starch and the majority of processed starchy foods (Bai et al., 2021; Wanikar & Kotwal, 2021). Damage to cells, tissues, and organs results from the significant stress induced by the blood glucose fluctuations that controls glucose homeostasis in the body (Bai et al., 2021). Although RS is not digested in the upper gastrointestinal tract, microbes connected to fermentation in the colon use it to make short‐chain fatty acids (SCFA). This gives the body more energy and contains a lot of butyrate, which is good for the health of the colon (Wanikar & Kotwal, 2021). An intermediate starch, the slowly digestible starch (SDS) is broken down gradually across the entire small intestine, resulting in a sustained release of glucose with a low initial glycemia and a subsequent slow and protracted release of glucose (Giacco & Brownlee, 2010). The current review revisits the effect of polyphenols like gallic acid and its ester derivatives on converting rapidly digestible starch to slowly digestible starch through starch–polyphenol complexation. Figure 2 shows the possible structures of formation of starch–gallic acid inclusion complexes.

The focus of the present review is to revisit the effect of natural polyphenols, particularly gallic acid and its ester derivatives on diabetes management. It will cover the effect of gallic acid and its derivatives on α‐glucosidase enzyme and also their effect on starch to make them slowly digestible through development of the inclusion complexes of the polyphenols in the starch core structure and their combined effect on enzymes responsible for Type II diabetes mellitus. Along with starch polyphenol inclusion efficacy in diabetes mellitus, this review will also throw light on the other diseases which can be controlled by the creation of the starch–polyphenol inclusion complex.

## 2 EFFECT OF STARCH ON HEALTH AND DISEASES

Starch is the most important source of calories in our bodies and is obtained from many staple foods like rice, millets, barley, wheat, and potatoes (Zhang & Hamaker, 2009). Starch is a homoglucan and has two main components, that is, amylose of a fundamentally linear molecule with limited branches, and amylopectin with many branches. Irrespective of the sources, starch consists of an alike backbone of repeating α‐D‐glucose units connected by α‐D‐(1 → 4) glycosidic linkages with branching points connected by αD‐(1 → 6) linkages. Amylose adopts a helical structure with a hydrophilic exterior and a hydrophobic interior in diluted solution, allowing it to interact with hydrophobic substances to create amylose‐inclusion complexes (Aller et al., 2011; Arijaje & Wang, 2017). RDS, like that found in white bread, is quickly digested and absorbed in the small intestine, which causes a sharp rise in blood sugar levels and subsequently a subsequent episode of hypoglycemia. This wide range in blood sugar puts a lot of stress on the system that controls glucose homeostasis, which can further cause damage to cells, tissues, and organs. As with raw potatoes and bananas, they go through microbial fermentation in the colon to produce short‐chain fatty acids (SCFA), which give the body more energy, as well as a lot of butyrate, which is good for colon health. With a low initial glycemia and a subsequent slow and extended release of glucose, SDS in raw cereal starch, is slowly digested across the entire small intestine to give sustained glucose release (Bai et al., 2021). Diabetes, also referred to as diabetes mellitus (DM), is a group of metabolic diseases that are characterized by hyperglycemia (high blood glucose levels for a prolonged period of time). This condition can be brought on by either insufficient insulin production by the pancreas or by cells that do not respond to the insulin that is produced (David & Gardner, 2011). Polydipsia (increased thirst), polyuria (frequent urination), and polyphagia are the three basic signs of high blood sugar (increased hunger). This chronic illness can have numerous implications if left untreated (Cooke & Plotnick, 2008). The most prevalent type of diabetes, Type 2 DM (T2DM), is characterized by insulin resistance, which may be coupled with somewhat lower insulin secretion (Ohkuwa et al., 1995). This causes hyperglycemia, which in turn causes the pancreatic β‐cells to malfunction. Long‐term hyperglycemia causes endogenous antioxidants to be impaired and produces more reactive oxygen species (ROS) (Ohkuwa et al., 1995). The deterioration of the pancreatic β‐cells and the development of diabetes sequelae such diabetes nephropathy (Cooke & Plotnick, 2008), diabetes retinopathy, and diabetes neuropathy (Hove et al., 2004) have all been linked to the oxidative stress brought on by the hyperglycemic state in T2DM. The potential of low glycemic index carbohydrates with high concentrations of resistant starch can modify diabetic nephropathy and prevent vitamin D deficiency which occurs due to the development of diabetes mellitus (Birt et al., 2013). A new study published in the journal of the American Heart Association has found out that eating starch‐rich snacks containing white potato increases the incidence of death by 50% and the increase in cardiovascular disease by 44%–57%. Conversely, eating foods rich in vegetables, fruits, and a low content of starch can lower the risk of cardiovascular‐related deaths and even diseases like cancer. Too much glucose in cancer is known to effect cancer metabolism by increasing the Warburg effect and generating precursors, such as amino acids, glucose, nucleotides, and fatty acids for uncontrolled proliferation of cancer cells (Han et al., 2017). Tumor cells are known to show an increase in glucose generation from starch, glycolysis, and gluconeogenesis (Bose & Le, 2018). A healthy gut microbiome is known to control glucose homeostasis, lactose digestion, bile acid generation, antioxidant enzyme upregulation, inflammation and blood lipid profile. However, an increase in the amount of starch and reduction in the levels of resistant starch can destroy the healthy gut microbiota, affecting health (Sanders et al., 2019). Water soluble fiber comprising of resistant starch and low amounts of starch is known to lower blood cholesterol and atherosclerosis by several mechanisms. Low quantities of starch are also known to increase fecal bulk content, hasten gastric emptying, shorten intestinal transit time, and promote digestive regularity (Soliman, 2019). Dietary fibers containing low amounts of starch produce short‐chain fatty acids in the large intestine, which act as anticarcinogenic agents and can reduce the chances of colorectal, small intestine, oral, larynx, and breast cancer (Lattimer & Haub, 2010).

## 3 POLYPHENOLS AND THEIR CHARACTERISTICS

Polyphenols are abundant in tea, chocolate, fruits and berries, vegetables, and other foods. Depending on how many phenol rings they have and what structural components hold those rings together, polyphenols can be divided into various classes. The primary classes are lignans, stilbenes, flavonoids, and phenolic acids. The risk of stroke, myocardial infarction, antiaging, diabetes, blood pressure, insulin resistance, and systemic inflammation has been reported to be lower in people who consume them. Resveratrol, a stilbene, and the flavonoid quercetin have also been connected to cardiometabolic health. Additionally, because gut bacteria convert polyphenols into bioactive molecules that have therapeutic effects, polyphenols can affect the composition of the gut microbiota, which is also independently linked to health benefits (Fraga et al., 2019). By modifying a variety of physiological processes, including cellular redox potential, enzymatic activity, cell proliferation, and signal transduction pathways, a polyphenol‐rich diet defends against chronic diseases. The paradox of high bioactivity and low bioavailability was brought to light by the fact that most polyphenols exhibited significant biological effects despite having poor oral bioavailability. The metabolites of polyphenols have garnered a lot of attention recently since several of them have biological effects that are comparable to or even superior to those of the parent substances (Luca et al., 2020). Additionally, naturally occurring polyphenols aid in the fight against the emergence of infections that are resistant to many drugs (Turuvekere Vittala Murthy et al., 2021). Gallic acid is a component of pharmaceuticals that have antioxidant, anticancer, antibacterial, chondro‐protective, carbonic anhydrase inhibitor, antidiabetic, anti‐ulcerogenic, and anti‐inflammatory properties (Al Zahrani et al., 2020). Gallic acid's anticancer effects result from a number of biological processes, including the induction of programmed cell death, cell cycle arrest, constriction of the vasculature, inhibition of tumor migration, and inflammation. Gallic acid is discovered to exhibit synergism with other chemotherapy medicines already in use (Tuli et al., 2022). Thus, gallic acid is a promising drug candidate for many diseases. Synergism with chemotherapeutic drugs would mean much fewer side effects from chemotherapeutic drugs because of the lower dosage of chemotherapy drug. Gallic acid and its derivatives (e.g., lipid‐soluble phenols such as synthetic gallic esters aka gallates) have been widely used in many therapeutic formulations, as an adjuvant, as a substitute for hydrocortisone in children with atopic dermatitis (AD), as a treatment for other skin conditions (hyperpigmentation, wound healing), and as a cosmetic ingredient. Gallic acid has been given the US Food and Drug Administration's GRAS status (generally recognized as safe), and it has shown to have relatively low systemic toxicity and related mortality at acute doses in several experimental models (Khan et al., 2018).

## 4 STARCH–POLYPHENOL INCLUSION COMPLEX IN FOOD TECHNOLOGY AND DISEASES

Known to generate inclusion complexes by creating hydrophobic–hydrophobic interactions with other hydrophobic moieties, such as colors, flavors, lactones, polymers, and lipids (Kumar & Loos, 2019), amylose chains of starch feature a helical hydrophobic interior cavity. The helical structure of amylose' hydrophilic moiety is oriented outward. Hydrophobic interactions are the main driving force behind V‐type starch–phenolic complexes (Rachmawati et al., 2015), which have been observed with ferulic acid, gallic acid, and green tea polyphenols. The digestibility of starch was only slightly reduced by complexation with caffeic acid, gallic acid, and ferulic acid with potato and maize amylopectin (Li, Pernell, & Ferruzzi, 2018). The starch from buckwheat was complexed with quercetin, which significantly reduced starch digestibility and increased crystallinity and compactness (Gao et al., 2021). By protecting them against oxidation, evaporation, and decomposition, the complex boosts the physical and chemical stability of hydrophobic molecules. For the creation of nanoencapsulation formations or the delayed and sustainable release of pharmaceuticals, certain types of inclusion complexes are necessary (Kumar & Loos, 2019). It was demonstrated in a different investigation on the development of quercetin complexes with amylose that these complexes effectively scavenged DPPH. Complexes demonstrated quercetin's significant retention in the stomach but sluggish release in the small intestine model. The amylose/quercetin complexes were found to be acceptable carriers for the regulated release of quercetin in the gastrointestinal system (Lv et al., 2019). When starch–TP complexes develop, tea polyphenols have been shown to inhibit amylose aggregation and starch retrogradation. As a result, starch granules’ gel strength decreased while their viscosity and gelatinization stability increased (Li, Zhai, et al., 2021). Thus, tea polyphenols can be used in food industrialization for preserving starch‐containing foods for a longer duration. In another report, formation of complexes of lotus seed with chlorogenic acid rendered the starch stronger and prevented the self‐assembly of starch. Thus, formation of these complexes can improve the storage and processing quality of lotus seed (Wang, Jiang, et al., 2021). Due to the development of complexes between them, green tea polyphenols had an inhibiting effect on the digestion of starch (Ayim et al., 2019). LSPs inhibited the hydrophobic interaction between normal maize starch and LSPs in a concentration‐dependent way, which also affected the rate and extent of regular maize starch digestion (longan seed polyphenols). This could lead to reduction of postprandial glycemic release upon formation of complexes and hence prove beneficial in diabetic patients (He et al., 2021). Chinese berry leaves heated with rice starch in aqueous solution made functional starch with slower digestion and hence could prove beneficial milieu in reducing sugar levels in the blood (Zheng et al., 2021). When starch and polyphenol molecules interacted, all of the starch molecules' propensities to aggregate were blocked, which decreased the relative crystallinity (RC) of the starch, flow resistance, and storage modulus and enhanced its water‐holding capacity. Thus, the retrogradation of starch was prevented. These findings are advantageous for the creation of high‐value, low‐retrogradation starch‐based products (Zeng et al., 2022). When potato starch was complexed with grape seed pro‐anthocyanidin, it included more resistant and slowly digestible starch than the original starch did (Zhang et al., 2020). Thus, it can reduce the glycemic load in diabetes mellitus and also prevent cardiovascular diseases. Similar to this, complexing wheat starch with young apple polyphenols improved intestinal health by increasing the concentration of short‐chain fatty acids (SCFAs), decreasing the glycemic load, and stimulating the formation of beneficial gut flora like *Prevotella* (Li, Yang, et al., 2021). Healthy gut microbiota and reduced glycemic load as have been reported by formation of starch polyphenol complexes can prevent diabetes, obesity (Schwarz et al., 2018), cardiovascular diseases (Schwarz et al., 2018), cancer (Schwarz et al., 2018), polycystic ovarian syndrome (Sharma et al., 2020), neurodegenerative disorders (Bergantin, 2019), and other inflammatory disorders. The changes in glucose metabolism often regulate tumor suppressors and oncogenes. A lot of therapies are being developed which target glucose uptake and glycolysis for inhibiting the cancer cells. Hence, reduced breakdown of starch in cancer patients can promote cancer cell apoptosis.

## 5 STARCH–GALLIC ACID INCLUSION COMPLEX IN DIABETES AND DISEASES

**FIGURE 3:**
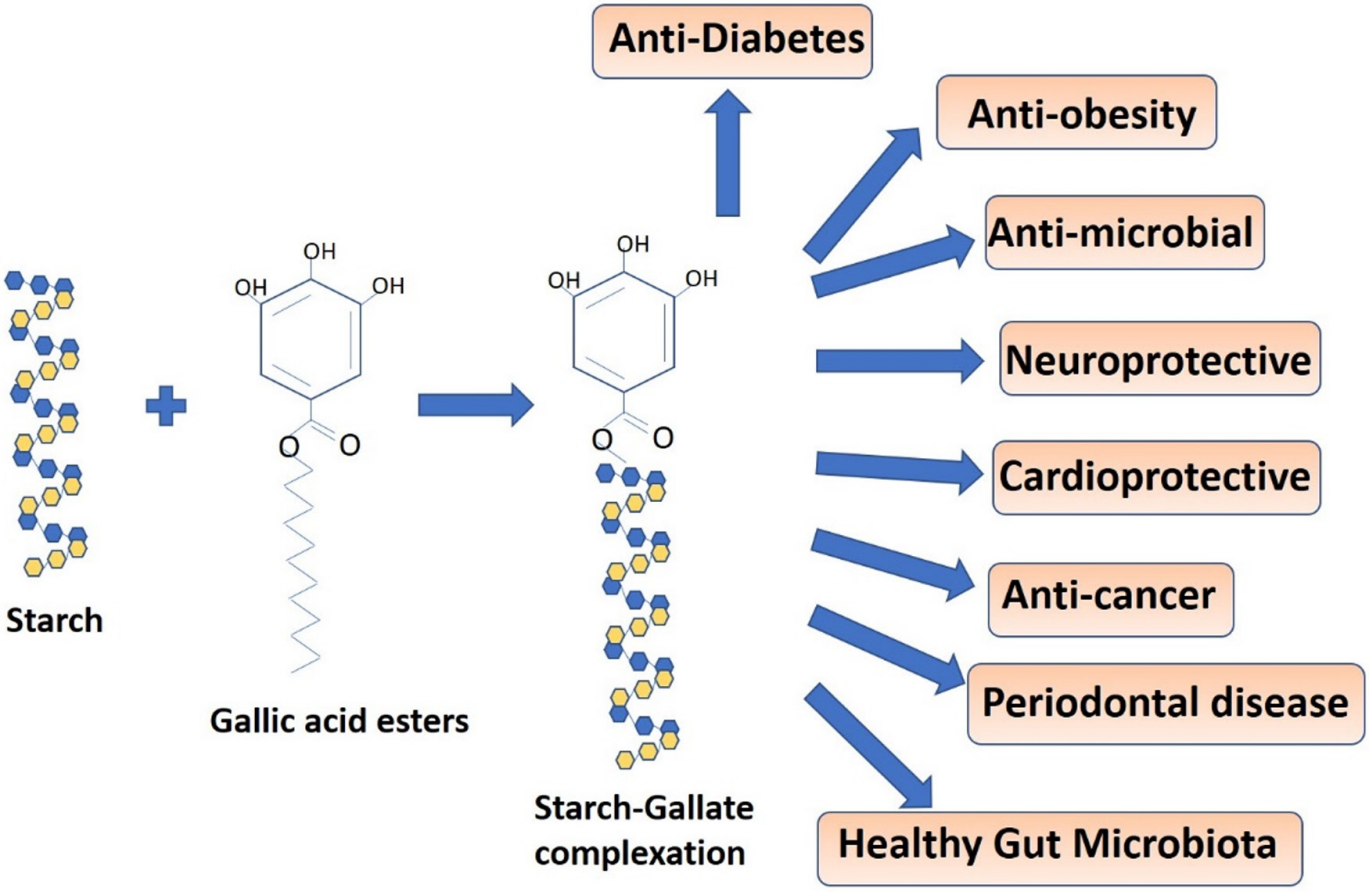
Activity of starch–polyphenol complexes in various disease control.

Gallic acid is tri hydroxybenzoic acid and it' is a phenolic acid. It is present in abundance in many plants and fruits, especially in gallnuts, sumac, witch hazel, tea leaves, oak bark, and other plants. It is a triphenolic compound with antioxidant, anti‐inflammatory, anti‐obesity, antidiabetic, antimicrobial, anticancer, anti‐myocardial ischemia properties. By using high‐pressure homogenization (HPH), rice starch and gallic acid were combined by van der Waals and hydrogen bonding forces to form a single complex. The complex structure in this was made more compact with the increased addition of gallic acid as a result of the increased rearrangement and aggregation behavior of the degraded starch molecular chains. This characteristic decreased the starch molecules’ accessibility to digestive enzymes, demonstrating the potential value of HPH and gallic acid complexation in regulating the digestion of starch products with desired digestibility (Liu et al., 2019). Another study found that gallic acid had higher binding affinities to α‐amylase than to starch chains and that gallic acid would non‐covalently interact with starch molecules and contribute to the creation of organized structures. As a result, during the hydrolysis process, gallic acid could be released from the complex and is more likely to do so by hydrogen bonds and van der Waals forces, occupying the active sites of Asp197, Asp300, His299, and Glu233 in the α‐amylase enzyme. This prevented starch from entering the active site pocket and decreased the digestibility of starch. These findings suggested that starch digestion behaviors and starch structure could be controlled through non‐covalent interactions between gallic acid and starch (Chi et al., 2017). Pea starch, on the other hand, requires less gallic acid to create a high level of complexation with a significant delay in starch digestion than do rice and maize starch (Villanova & Lin, 2022). Complexing pea starch, maize starch, and rice starch with gallic acid in the above references has been shown to improve glycemic indices in diabetic patients by causing a significant delay in starch digestion. It could lead to reduction of postprandial glycemic release in diabetic patients (He et al., 2021; Zheng et al., 2021). Complexing gallic acid with hydroxypropyl‐β‐cyclodextrin, a chemically modified cyclic oligosaccharide made from starch, increased the solubility of gallic acid for the treatment of *Candida albicans*. Thus, complex formation can increase the antifungal capacity of gallic acid (Teodoro et al., 2017). Gallic acid was complexed with corn starch in another report to increase the quality of food (Tan et al., 2022). Complexing cyclodextrin, a polysaccharide obtained from the degradation of starch with gallic acid and encapsulating them in polylactic acid nanofibers increased the antioxidant potential of gallic acid, and therefore, this idea would be explored to increase the freshness and shelf life of food (Aytac et al., 2016). Phenolic compounds (gallic acid, catechin, and epigallocatechin gallate) are degraded less in the presence of starch as compared with the presence of starch–protein complex (Ferruzzi et al., 2020). Thus, we can say that starch forms a much stronger complex with polyphenols and a higher affinity of polyphenols to starch as compared with starch–protein complex. Gallic acid was added to maize and potato starch, which had no chemical changes but dramatically lowered the glycemic indices and gelatinization temperatures (*p* < .05). Gallic acid may bind to starch by non‐covalent CH‐π bonds along α‐(1 4) glycosidic chains, as suggested by the significantly lower levels of phenolic proton intensities and hydrodynamic radii in starch–phenolic complexes than in control starch–phenolic mixes (*p* < .05) (Li et al., 2020). Upon complexing propyl gallate with beta cyclodextrin, a cyclic derivative of starch, the water solubility of propyl gallate increased and also the complex formation lead to increase in the antioxidant activity (free hydroxyl radical and superoxide radical scavenging), thus protecting from the diseases which occur due to the generation of superoxide radicals (Li, Pu, et al., 2018). Binding of dodecyl gallate, a derivative of polyphenol gallic acid, to rice starch increased the concentration of slowly digestible starch, resistant starch in the intestine with potent antioxidant benefits and better digestion (Chi et al., 2018). Hence, binding of gallic acid or their derivatives to starch increases the antioxidant potential of gallic acid and can play a preventive role in many diseases especially diabetes. Series of alkyl gallates upon binding to beta cyclodextrin has been reported have antibacterial activity in Gram‐negative foodborne bacteria (*Pseudomonas fluorescens* and *Vibrio parahaemolyticus*). Length of the alkyl chain plays a very important role in mediating efficient antibacterial action, especially octyl gallate (Shi et al., 2022). Greater length of alkyl chains has damaged the bacterial membrane and destroyed the respiratory electron transport chain to a much greater extent than short alkyl chains. Increased length of alkyl chains as in ethyl, propyl, octyl or dodecyl gallate (gallic acid derivatives) forming inclusion complex with starch not only lowers the rate of digestion of starch, but also the effect of gallic acid inhibits the enzyme alpha glucosidase. Gallic acid can increase the insulin uptake by activating the PPAR‐γ, Akt, and AMPK pathways. The expression of TNF‐ and adipocytokines may also be controlled to enhance gallic acid’ antidiabetic effects. By inhibiting β‐cell death, gallic acid enhanced the function of the cells. Starch gallic acid complex could revert glyoxal‐induced renal cell death, membrane rupture, ROS generation, lipid peroxidation, mitochondrial membrane potential collapse, and lysosomal membrane discharge, which lead to advanced glycation inhibition. Thus, formation of starch–gallic acid complex has a dual role in preventing diabetes (Visvanathan et al., 2021). Epigallocatechin gallate (EGCG) upon complexing with starch, especially amylose inhibits alpha amylase enzyme (Xu et al., 2021). The function of alpha amylase is breaking down starch into glucose. Also, gallic acid inclusion complex with starch upregulates glucose transporter 2 (GLUT2) and glycogen synthase 2 (GYS2) and downregulates the expression of gluconeogenic genes (Krishnan et al., 2021). Therefore, inhibition of alpha amylase (Sales et al., 2012) and gluconeogenic genes and upregulation of glucose transporter 2 and glycogen synthase 2 will lead to decrease of blood glucose concentration, and thereby serve as an effective molecule in diabetes, obesity, dental caries, and periodontal diseases. Beta cyclodextrin, an enzymatic modification of starch when forms inclusion complex with gallic acid improves the thermal stability, storage stability, antiglycation and antioxidant potential of gallic acid (Ntuli et al., 2022). There are previous reports which suggest that increased blood glucose levels lead to increase in disease progression for cancer, cardiac failure, and obesity. Hence, various nclusion complexes of starch with gallic acid can prove to be anticarcinogenic, anti‐obesity, antimicrobial, neuro protective, and cardioprotective in nature. It can reduce the atmospheric and photooxidation of gallic acid, thereby reducing its rate of degradation and leading to a slow and sustainable release. Figure 3 depicts various diseases controlled by the formation of starch–gallic acid ester complexes.

## 6 METHODOLOGY FOR THE PREPARATION OF STARCH–GALLIC ACID INCLUSION COMPLEX

Rice starch gallic acid inclusion complex was prepared by high‐pressure homogenization technique. In this, the rice starch slurry was prepared and then heated in water bath at 95°C for half an hour. Gallic acid was added to this paste while continuous stirring and the mixtures were made uniform in a Nano High‐Pressure Homogenizer at 150 MPa, thrice. The precipitates were obtained by centrifugation and washed with 50% ethanol to remove unbound gallic acid. These precipitates were freeze‐dried and grinded with laboratory crusher to obtain the final powder of rice starch–gallic acid complex (Liu et al., 2019). Pea starch was complexed with starch using a similar high‐pressure homogenization technique (Villanova & Lin, 2022). For maize starch–gallic acid complex, starch was suspended in distilled water with constant stirring followed by the addition of gallic acid at a pH of 4 and at different molar concentrations. A gentle nitrogen stream was used to effervesce the slurry for 30 minutes while shearing it at a high speed of 6000 rpm. The starch–gallic acid complex slurry was then adjusted to a pH of 7.0, washed three times with distilled water, and allowed to air dry at 40°C (Chi et al., 2017). The unbound fraction of gallic acid can be determined by folin ciocalteu's method (Chi et al., 2017). Potato starch can be suspended in phenolic acids and dispersed in hydrochloric acid followed by centrifugation and freeze‐drying to obtain powder (Li et al., 2020). For gallic acid and hydroxypropyl‐β‐cyclodextrin (enzymatically modified starch) inclusion complex preparation, gallic acid and HPβCD were mixed in distilled water and passed through a spray drier. The total percentage of gallic acid and modified starch in the complex was determined by HPLC (Teodoro et al., 2017). However, in another study, starch–HPβCD complexes can be prepared by the slurry method, by mixing starch and HPβCD in double‐distilled water and keeping in hood to obtain powder (Aytac et al., 2016). For preparing the inclusion complex of starch–long chain derivatives of gallate, propyl gallate and starch were mixed in 40% ethanol solution, stirred for 6 hrs and powder was obtained by freeze‐drying (Li, Pu, et al., 2018). Similarly, for the preparation of rice starch–dodecyl gallate complex, gelatinized starch would be added to different concentrations of dodecyl gallate, washed with 70% ethanol and will be air‐dried and ground to fresh powder (Chi et al., 2018). Therefore, the standard preparation module of the starch– polyphenol inclusion complex is mostly simple in nature and can be prepared readily under any standard laboratory set up and conditions.

## 7 CHARACTERIZATION OF STARCH–GALLIC ACID INCLUSION COMPLEX

To study the crystalline structure of starch and starch–gallic acid inclusion complexes, certain X‐Ray Diffraction patterns were observed. Upon the addition of gallic acid to rice starch, V‐type diffraction shapes with distinguishing peaks at 2*θ* = 13.0° and 19.8° (typical of V7 helical type diffraction) were observed. The concentration of gallic acid distorted the comparative peak intensities of XRD for the development of the starch–gallic acid inclusion complex (Liu et al., 2019). The unique peaks of gallic acid disappeared after high‐pressure homogenization of the starch–gallic acid inclusion complex, demonstrating that some molecular chains of rice starch interacted with gallic acid to create a V‐helical structure, shielding the characteristic diffraction peak of gallic acid. Also upon complex formation for rice starch (Liu et al., 2019), pea starch (Villanova & Lin, 2022) and maize starch (Chi et al., 2017)–gallic acid inclusion complex, with the growth in addition gallic acid, the relative crystallinity of inclusion complex is augmented. Fractal geometry demonstrated that gallic acid increased the compactness of starch scattering objects following HPH treatment. Additional peaks that were attributed to carbonyl (C=O) vibrations and stretching vibrations of the phenyl ring of gallic acid appeared after the formation of the starch–gallic acid inclusion combination. For rice starch, maize starch, and pea starch–gallic acid inclusion complex intensity of the peaks also increased upon increasing concentration of gallic acid (Chi et al., 2017; Liu et al., 2019; Villanova & Lin, 2022). The starch carbons C1 and C4 in the nuclear magnetic resonance spectra showed a noticeable alteration, but the peaks carbons C2,3,5,6 were not noticeably changed upon formation of the starch–gallic acid inclusion complex. Inclusion complex showed more resolved spectra than starch alone. Also NMR confirmed the formation of V‐type single‐helical structure, which is consistent with XRD analysis (Liu et al., 2019).

## 8 CONCLUSION

The review highlights that the preparation and application of starch–gallic acid inclusion complexes would prove very beneficial in ameliorating many metabolic disorders, such as diabetes mellitus, atherosclerosis, cardiovascular disease, cholesterol, digestive disorders, cancer, periodontal diseases, and other diseases which occur on account of an increase in starch hydrolysis and increment in blood glucose levels. Therefore, this review will be beneficial to many food technologists for food modifications and pharmacists for synthesizing drugs encompassing starch–gallic acid inclusion complexes, necessary for applications in diet and in diseases.

## CONFLICT OF INTEREST

Authors state that there are no conflicts of interest in publication of this review.

## Data Availability

The data that support the findings of this study are available on request from the corresponding author. The data are not publicly available due to privacy or ethical restrictions.

## References

[fsn33208-bib-0001] Al Zahrani, N. A. , El‐Shishtawy, R. M. , & Asiri, A. M. (2020, October 15). Recent developments of gallic acid derivatives and their hybrids in medicinal chemistry: A review. European Journal of Medicinal Chemistry, 204, 112609. 10.1016/j.ejmech.2020.112609 32731188

[fsn33208-bib-0002] Aller, E. E. , Abete, I. , Astrup, A. , Martinez, J. A. , & van Baak, M. A. (2011, March). Starches, sugars and obesity. Nutrients, 3(3), 341–369. 10.3390/nu3030341 22254101 PMC3257742

[fsn33208-bib-0003] Alssema, M. , Boers, H. M. , Ceriello, A. , Kilpatrick, E. S. , Mela, D. J. , Priebe, M. G. , Schrauwen, P. , Wolffenbuttel, B. H. , & Pfeiffer, A. F. (2015, January 28). Diet and glycaemia: The markers and their meaning. A report of the Unilever Nutrition Workshop. The British Journal of Nutrition, 113(2), 239–248. 10.1017/S0007114514003547 25498786 PMC4302387

[fsn33208-bib-0004] Arijaje, E. O. , & Wang, Y.‐J. (2017). Effects of chemical and enzymatic modifications on starch‐linoleic acid complex formation. Food Chemistry, 217, 9–17 ISSN 0308‐8146. 27664602 10.1016/j.foodchem.2016.08.072

[fsn33208-bib-0005] Ayim, I. , Ma, H. , Alenyorege, E. A. , & Duan, Y. (2019). In vitro inhibitory effect of tea extracts on starch digestibility. Journal of Food Process Engineering, 42(4), 0145–8876.

[fsn33208-bib-0006] Aytac, Z. , Kusku, S. I. , Durgun, E. , & Uyar, T. (2016, June). Encapsulation of gallic acid/cyclodextrin inclusion complex in electrospun polylactic acid nanofibers: Release behavior and antioxidant activity of gallic acid. Materials Science & Engineering. C, Materials for Biological Applications, 63, 231–239. 10.1016/j.msec.2016.02.063 27040215

[fsn33208-bib-0007] Bai, Y. , Li, X. , Ji, H. , Yu, W. , Zheng, D. , Wang, Y. , & Jin, Z. (2021). A review of the design and architecture of starch‐based dietary foods. Engineering, 7(5), 663–673. 10.1016/j.eng.2020.12.007

[fsn33208-bib-0008] Bao, J. (2017). Starch in health and disease. Starch ‐ Stärke, 69, 1770076. 10.1002/star.201770076

[fsn33208-bib-0009] Berbudi, A. , Rahmadika, N. , Tjahjadi, A. I. , & Ruslami, R. (2020). Type 2 diabetes and its impact on the immune system. Current Diabetes Reviews, 16(5), 442–449. 10.2174/1573399815666191024085838 31657690 PMC7475801

[fsn33208-bib-0010] Bergantin, L. B. (2019). Hypertension, diabetes and neurodegenerative diseases: Is there a clinical link through the Ca2+/cAMP signalling interaction? Current Hypertension Reviews, 15(1), 32–39. 10.2174/1573402114666180817113242 30117399

[fsn33208-bib-0011] Birt, D. F. , Boylston, T. , Hendrich, S. , Jane, J. L. , Hollis, J. , Li, L. , McClelland, J. , Moore, S. , Phillips, G. J. , Rowling, M. , Schalinske, K. , Scott, M. P. , & Whitley, E. M. (2013, November 6). Resistant starch: Promise for improving human health. Advances in Nutrition, 4(6), 587–601. 10.3945/an.113.004325 24228189 PMC3823506

[fsn33208-bib-0012] Bose, S. , & Le, A. (2018). Glucose metabolism in cancer. Advances in Experimental Medicine and Biology, 1063, 3–12. 10.1007/978-3-319-77736-8_1 29946772

[fsn33208-bib-0013] Chi, C. , Li, X. , Feng, T. , Zeng, X. , Chen, L. , & Li, L. (2018, September 5). Improvement in nutritional attributes of rice starch with dodecyl gallate complexation: A molecular dynamic simulation and in vitro study. Journal of Agricultural and Food Chemistry, 66(35), 9282–9290. 10.1021/acs.jafc.8b02121 30114360

[fsn33208-bib-0014] Chi, C. , Li, X. , Zhang, Y. , Chen, L. , Li, L. , & Wang, Z. (2017, February 22). Digestibility and supramolecular structural changes of maize starch by non‐covalent interactions with gallic acid. Food & Function, 8(2), 720–730. 10.1039/c6fo01468b 28106222

[fsn33208-bib-0015] Chung, H.‐J. , Donner, E. , & Liu, Q. (2011). Resistant starches in foods. In M. Moo‐Young (Ed.), Comprehensive biotechnology (2nd ed., pp. 527–534). ISBN 9780080885049. Academic Press. 10.1016/B978-0-08-088504-9.00309-3

[fsn33208-bib-0016] Cooke, D. W. , & Plotnick, L. (2008). Type 1 diabetes mellitus in pediatrics. Pediatrics in Review, 29, 374–384. 18977856 10.1542/pir.29-11-374

[fsn33208-bib-0017] David, G. , & Gardner, D. (2011). Greenspan's basic & clinical endocrinology (9th ed.). McGraw‐Hill Medical.

[fsn33208-bib-0018] Feingold, K. R. (2000). Oral and injectable (non‐insulin) pharmacological agents for the treatment of type 2 diabetes. In K. R. Feingold , B. Anawalt , A. Boyce , G. Chrousos , W. W. de Herder , K. Dhatariya , K. Dungan , J. M. Hershman , J. Hofland , S. Kalra , G. Kaltsas , C. Koch , P. Kopp , M. Korbonits , C. S. Kovacs , W. Kuohung , B. Laferrère , M. Levy , E. A. McGee , et al. (Eds.), Endotext [Internet]. MDText.com, Inc. https://www.ncbi.nlm.nih.gov/books/NBK279141/ 25905364

[fsn33208-bib-0019] Ferruzzi, M. G. , Hamaker, B. R. , & Bordenave, N. (2020, March 30). Phenolic compounds are less degraded in presence of starch than in presence of proteins through processing in model porridges. Food Chemistry, 309, 125769. 10.1016/j.foodchem.2019.125769 31734007

[fsn33208-bib-0020] Forester, S. C. , Gu, Y. , & Lambert, J. D. (2012, November). Inhibition of starch digestion by the green tea polyphenol, (‐)‐epigallocatechin‐3‐gallate. Molecular Nutrition & Food Research, 56(11), 1647–1654. 10.1002/mnfr.201200206 23038646 PMC3683549

[fsn33208-bib-0021] Fraga, C. G. , Croft, K. D. , Kennedy, D. O. , & Tomás‐Barberán, F. A. (2019, February 20). The effects of polyphenols and other bioactives on human health. Food & Function, 10(2), 514–528. 10.1039/c8fo01997e 30746536

[fsn33208-bib-0022] Gao, S. , Liu, H. , Sun, L. , Cao, J. , Yang, J. , Lu, M. , & Wang, M. (2021). Rheological, thermal and in vitro digestibility properties on complex of plasma modified Tartary buckwheat starches with quercetin. Food Hydrocolloids, 110, 106209. 10.1016/j.foodhyd.2020.106209

[fsn33208-bib-0023] Giacco, F. , & Brownlee, M. (2010, October 29). Oxidative stress and diabetic complications. Circulation Research, 107(9), 1058–1070. 10.1161/CIRCRESAHA.110.223545 21030723 PMC2996922

[fsn33208-bib-0024] Han, H. , Zhang, T. , Jin, Z. , Guo, H. , Wei, X. , Liu, Y. , Chen, Q. , & He, J. (2017, July 25). Blood glucose concentration and risk of liver cancer: Systematic review and meta‐analysis of prospective studies. Oncotarget, 8(30), 50164–50173. 10.18632/oncotarget.16816 28432278 PMC5564840

[fsn33208-bib-0025] He, T. , Wang, K. , Zhao, L. , Chen, Y. , Zhou, W. , Liu, F. , & Hu, Z. (2021, March 15). Interaction with longan seed polyphenols affects the structure and digestion properties of maize starch. Carbohydrate Polymers, 256, 117537. 10.1016/j.carbpol.2020.117537 33483053

[fsn33208-bib-0026] Hove, M. N. , Kristensen, J. K. , Lauritzen, T. , & Bek, T. (2004). The prevalence of retinopathy in an unselected population of type‐2 diabetes patients from Arhus County, Denmark. Acta Ophthalmologica Scandinavica, 82, 443–448. 15291939 10.1111/j.1600-0420.2004.00270.x

[fsn33208-bib-0027] Khan, B. A. , Mahmood, T. , Menaa, F. , Shahzad, Y. , Yousaf, A. M. , Hussain, T. , & Ray, S. D. (2018). New perspectives on the efficacy of gallic acid in cosmetics & nanocosmeceuticals. Current Pharmaceutical Design, 24(43), 5181–5187. 10.2174/1381612825666190118150614 30657034

[fsn33208-bib-0028] Krishnan, V. , Rani, R. , Awana, M. , Pitale, D. , Kulshreshta, A. , Sharma, S. , Bollinedi, H. , Singh, A. , Singh, B. , Singh, A. K. , & Praveen, S. (2021, January 15). Role of nutraceutical starch and proanthocyanidins of pigmented rice in regulating hyperglycemia: Enzyme inhibition, enhanced glucose uptake and hepatic glucose homeostasis using in vitro model. Food Chemistry, 335, 127505. 10.1016/j.foodchem.2020.127505 32739823

[fsn33208-bib-0029] Kumar, K. , & Loos, K. (2019). Deciphering structures of inclusion complexes of amylose with natural phenolic amphiphiles. ACS Omega, 4(18), 17807–17813. 10.1021/acsomega.9b02388 31681887 PMC6822131

[fsn33208-bib-0031] Lattimer, J. M. , & Haub, M. D. (2010, December). Effects of dietary fiber and its components on metabolic health. Nutrients, 2(12), 1266–1289. 10.3390/nu2121266 22254008 PMC3257631

[fsn33208-bib-0032] Li, D. , Yang, Y. , Yang, X. , Wang, X. , Guo, C. , Sun, L. , & Guo, Y. (2021, March 7). Modulation of gelatinized wheat starch digestion and fermentation profiles by young apple polyphenols in vitro. Food & Function, 12(5), 1983–1995. 10.1039/d0fo02752a 33537688

[fsn33208-bib-0033] Li, H. , Zhai, F. , Li, J. , Zhu, X. , Guo, Y. , Zhao, B. , & Xu, B. (2021, January 1). Physicochemical properties and structure of modified potato starch granules and their complex with tea polyphenols. International Journal of Biological Macromolecules, 166, 521–528. 10.1016/j.ijbiomac.2020.10.209 33129907

[fsn33208-bib-0034] Li, M. , Ndiaye, C. , Corbin, S. , Foegeding, E. A. , & Ferruzzi, M. G. (2020, March 5). Starch‐phenolic complexes are built on physical CH‐π interactions and can persist after hydrothermal treatments altering hydrodynamic radius and digestibility of model starch‐based foods. Food Chemistry, 308, 125577. 10.1016/j.foodchem.2019.125577 31669942

[fsn33208-bib-0035] Li, M. , Pernell, C. , & Ferruzzi, M. G. (2018). Complexation with phenolic acids affect rheological properties and digestibility of potato starch and maize amylopectin. Food Hydrocolloids, 77, 843–852. 10.1016/j.foodhyd.2017.11.028

[fsn33208-bib-0036] Li, Q. , Pu, H. , Tang, P. , Tang, B. , Sun, Q. , & Li, H. (2018, April 15). Propyl gallate/cyclodextrin supramolecular complexes with enhanced solubility and radical scavenging capacity. Food Chemistry, 245, 1062–1069. 10.1016/j.foodchem.2017.11.065 29287323

[fsn33208-bib-0037] Liu, Y. , Chen, L. , Xu, H. , Liang, Y. , & Zheng, B. (2019, August 1). Understanding the digestibility of rice starch‐gallic acid complexes formed by high pressure homogenization. International Journal of Biological Macromolecules, 134, 856–863. 10.1016/j.ijbiomac.2019.05.083 31103593

[fsn33208-bib-0038] Luca, S. V. , Macovei, I. , Bujor, A. , Miron, A. , Skalicka‐Woźniak, K. , Aprotosoaie, A. C. , & Trifan, A. (2020). Bioactivity of dietary polyphenols: The role of metabolites. Critical Reviews in Food Science and Nutrition, 60(4), 626–659. 10.1080/10408398.2018.1546669 30614249

[fsn33208-bib-0039] Lv, R. , Liang, Q. , Zou, Y. , Zou, J. , Luo, Z. , Shao, P. , & Tamer, T. M. (2019). Preparation and structural properties of amylose complexes with quercetin and their preliminary evaluation in delivery application. International Journal of Food Properties, 22(1), 1445–1462. 10.1080/10942912.2019.1651736

[fsn33208-bib-0040] Mah, E. , Garcia‐Campayo, V. , & Liska, D. (2018, August 9). Substitution of corn starch with resistant starch type 4 in a breakfast bar decreases postprandial glucose and insulin responses: A randomized, controlled, crossover study. Current Developments in Nutrition, 2(10), nzy066. 10.1093/cdn/nzy066 30338311 PMC6186909

[fsn33208-bib-0041] Malunga, L. N. , Eck, P. , & Beta, T. (2016). Inhibition of intestinal α‐glucosidase and glucose absorption by feruloylated arabinoxylan mono‐ and oligosaccharides from corn bran and wheat aleurone. Journal of Nutrition and Metabolism, 2016, 1932532. 10.1155/2016/1932532 27073693 PMC4814672

[fsn33208-bib-0042] Miao, M. , Jiang, B. , Cui, S. W. , Zhang, T. , & Jin, Z. (2015). Slowly digestible starch—A review. Critical Reviews in Food Science and Nutrition, 55(12), 1642–1657. 10.1080/10408398.2012.704434 24915311

[fsn33208-bib-0043] Ntuli, S. , Leuschner, M. , Bester, M. J. , & Serem, J. C. (2022, June 14). Stability, morphology, and effects of in vitro digestion on the antioxidant properties of polyphenol inclusion complexes with β‐cyclodextrin. Molecules, 27(12), 3808. 10.3390/molecules27123808 35744933 PMC9228204

[fsn33208-bib-0044] Ohkuwa, T. , Sato, Y. , & Naoi, M. (1995). Hydroxyl radical formation in diabetic fats induced by streptozotocin. Life Sciences, 56, 1789–1798. 7739353 10.1016/0024-3205(95)00150-5

[fsn33208-bib-0045] Omage, K. , & Omage, S. O. (2017, November 24). Evaluation of the glycemic indices of three commonly eaten mixed meals in Okada, Edo State. Food Science & Nutrition, 6(1), 220–228. 10.1002/fsn3.550 29387382 PMC5778211

[fsn33208-bib-0046] Rachmawati, R. , Woortman, A. J. J. , Kumar, K. , & Loos, K. (2015). Inclusion complexes between polytetrahydrofuran‐b‐amylose block copolymers and polytetrahydrofuran chains. Macromolecular Bioscience, 15, 812–828. 10.1002/mabi.201400515 25706353

[fsn33208-bib-0047] Rorsman, P. , & Ashcroft, F. M. (2018, January 1). Pancreatic β‐cell electrical activity and insulin secretion: Of mice and men. Physiological Reviews, 98(1), 117–214. 10.1152/physrev.00008.2017 29212789 PMC5866358

[fsn33208-bib-0048] Sales, P. M. , Souza, P. M. , Simeoni, L. A. , & Silveira, D. (2012). α‐Amylase inhibitors: A review of raw material and isolated compounds from plant source. Journal of Pharmacy & Pharmaceutical Sciences, 15(1), 141–183. 10.18433/j35s3k 22365095

[fsn33208-bib-0049] Sanders, M. E. , Merenstein, D. J. , Reid, G. , Gibson, G. R. , & Rastall, R. A. (2019, October). Probiotics and prebiotics in intestinal health and disease: From biology to the clinic. Nature Reviews. Gastroenterology & Hepatology, 16(10), 605–616. 10.1038/s41575-019-0173-3 Erratum in: Nature Reviews Gastroenterology & Hepatology. 2019, August 9. 31296969

[fsn33208-bib-0050] Schwarz, P. E. H. , Timpel, P. , Harst, L. , Greaves, C. J. , Ali, M. K. , Lambert, J. , Weber, M. B. , Almedawar, M. M. , & Morawietz, H. (2018, October 9). Blood sugar regulation for cardiovascular health promotion and disease prevention: JACC health promotion series. Journal of the American College of Cardiology, 72(15), 1829–1844. 10.1016/j.jacc.2018.07.081 30286928 PMC6709577

[fsn33208-bib-0051] Sharma, Y. K. , Chauhan, S. , Singh, P. , & Deo, K. (2020, May 10). Correlation of cutaneous manifestations with body mass index, blood glucose, and hormonal levels in patients with polycystic ovarian disease. Indian Dermatology Online Journal, 11(3), 378–381. 10.4103/idoj.IDOJ_193_18 32695697 PMC7367582

[fsn33208-bib-0052] Shi, Y. G. , Li, D. H. , Kong, Y. M. , Zhang, R. R. , Gu, Q. , Hu, M. X. , Tian, S. Y. , & Jin, W. G. (2022, January 16). Enhanced antibacterial efficacy and mechanism of octyl gallate/beta‐cyclodextrins against Pseudomonas fluorescens and Vibrio parahaemolyticus and incorporated electrospun nanofibers for Chinese giant salamander fillets preservation. International Journal of Food Microbiology, 361, 109460. 10.1016/j.ijfoodmicro.2021.109460 34785387

[fsn33208-bib-0053] Soliman, G. A. (2019, May 23). Dietary fiber, atherosclerosis, and cardiovascular disease. Nutrients, 11(5), 1155. 10.3390/nu11051155 31126110 PMC6566984

[fsn33208-bib-0054] Tan, J. , Li, P. , Wang, W. , Cai, X. , & Xue, H. (2022, May 31). Separation of gallic acid from *Cornus officinalis* and its interactions with corn starch. International Journal of Biological Macromolecules, 208, 390–399. 10.1016/j.ijbiomac.2022.03.116 35339498

[fsn33208-bib-0055] Teodoro, G. R. , Gontijo, A. V. L. , Borges, A. C. , Tanaka, M. H. , Lima, G. M. G. , Salvador, M. J. , & Koga‐Ito, C. Y. (2017, July 11). Gallic acid/hydroxypropyl‐β‐cyclodextrin complex: Improving solubility for application on in vitro/ in vivo Candida albicans biofilms. PLoS One, 12(7), e0181199. 10.1371/journal.pone.0181199 28700692 PMC5507443

[fsn33208-bib-0056] Tomic, D. , Shaw, J. E. , & Magliano, D. J. (2022, September). The burden and risks of emerging complications of diabetes mellitus. Nature Reviews. Endocrinology, 18(9), 525–539. 10.1038/s41574-022-00690-7 PMC9169030 35668219

[fsn33208-bib-0057] Tuli, H. S. , Mistry, H. , Kaur, G. , Aggarwal, D. , Garg, V. K. , Mittal, S. , Yerer, M. B. , Sak, K. , & Khan, M. A. (2022). Gallic acid: A dietary polyphenol that exhibits anti‐neoplastic activities by modulating multiple oncogenic targets. Anti‐Cancer Agents in Medicinal Chemistry, 22(3), 499–514. 10.2174/1871520621666211119085834 34802408

[fsn33208-bib-0058] Turuvekere Vittala Murthy, N. , Agrahari, V. , & Chauhan, H. (2021, April). Polyphenols against infectious diseases: Controlled release nano‐formulations. European Journal of Pharmaceutics and Biopharmaceutics, 161, 66–79. 10.1016/j.ejpb.2021.02.003 33588032

[fsn33208-bib-0059] Villanova, F. A. , & Lin, A. H. (2022, June 28). Modification of pea starch digestibility through the complexation with gallic acid via high‐pressure homogenization. Polymers (Basel), 14(13), 2623. 10.3390/polym14132623 35808669 PMC9269514

[fsn33208-bib-0060] Visvanathan, R. , Houghton, M. J. , & Williamson, G. (2021, May 1). Maltoheptaoside hydrolysis with chromatographic detection and starch hydrolysis with reducing sugar analysis: Comparison of assays allows assessment of the roles of direct α‐amylase inhibition and starch complexation. Food Chemistry, 343, 128423. 10.1016/j.foodchem.2020.128423 33168261

[fsn33208-bib-0061] Wang, J. , Jiang, X. , Zheng, B. , & Zhang, Y. (2021, November). Structural and physicochemical properties of lotus seed starch‐chlorogenic acid complexes prepared by microwave irradiation. Journal of Food Science and Technology, 58(11), 4157–4166. 10.1007/s13197-020-04881-w 34538900 PMC8405777

[fsn33208-bib-0062] Wang, Y. , Alkhalidy, H. , & Liu, D. (2021, January 29). The emerging role of polyphenols in the management of type 2 diabetes. Molecules, 26(3), 703. 10.3390/molecules26030703 33572808 PMC7866283

[fsn33208-bib-0063] Wanikar, R. , & Kotwal, S. (2021). Resistant starch: A promising functional food ingredient. In M. O. Emeje (Ed.), Starch—Evolution and recent advances [Internet]. IntechOpen. 10.5772/intechopen.101558 https://www.intechopen.com/chapters/79847

[fsn33208-bib-0064] Xu, Y. , Tang, G. , Zhang, C. , Wang, N. , & Feng, Y. (2021, November 24). Gallic acid and diabetes mellitus: Its association with oxidative stress. Molecules, 26(23), 7115. 10.3390/molecules26237115 34885698 PMC8658971

[fsn33208-bib-0065] Zeng, X. , Zheng, B. , Xiao, G. , & Chen, L. (2022, January 15). Synergistic effect of extrusion and polyphenol molecular interaction on the short/long‐term retrogradation properties of chestnut starch. Carbohydrate Polymers, 276, 118731. 10.1016/j.carbpol.2021.118731 34823767

[fsn33208-bib-0066] Zhang, G. , & Hamaker, B. R. (2009, November). Slowly digestible starch: Concept, mechanism, and proposed extended glycemic index. Critical Reviews in Food Science and Nutrition, 49(10), 852–867. 10.1080/10408390903372466 19960393

[fsn33208-bib-0067] Zhang, Z. , Tian, J. , Fang, H. , Zhang, H. , Kong, X. , Wu, D. , Zheng, J. , Liu, D. , Ye, X. , & Chen, S. (2020, March 3). Physicochemical and digestion properties of potato starch were modified by complexing with grape seed proanthocyanidins. Molecules, 25(5), 1123. 10.3390/molecules25051123 32138212 PMC7179102

[fsn33208-bib-0068] Zheng, Y. , Tian, J. , Kong, X. , Wu, D. , Chen, S. , Liu, D. , & Ye, X. (2021, January 15). Proanthocyanidins from Chinese berry leaves modified the physicochemical properties and digestive characteristic of rice starch. Food Chemistry, 335, 127666. 10.1016/j.foodchem.2020.127666 32739821

